# cuteSV-OL: a real-time structural variation detection framework for nanopore sequencing devices

**DOI:** 10.1093/bioinformatics/btaf668

**Published:** 2025-12-17

**Authors:** Weimin Guo, Yadong Liu, Yadong Wang, Tao Jiang

**Affiliations:** Center for Bioinformatics, Faculty of Computing, Harbin Institute of Technology, Harbin, Heilongjiang 150001, China; Center for Bioinformatics, Faculty of Computing, Harbin Institute of Technology, Harbin, Heilongjiang 150001, China; Zhengzhou Research Institute, Harbin Institute of Technology, Zhengzhou, Henan 450000, China; Center for Bioinformatics, Faculty of Computing, Harbin Institute of Technology, Harbin, Heilongjiang 150001, China; Zhengzhou Research Institute, Harbin Institute of Technology, Zhengzhou, Henan 450000, China; Center for Bioinformatics, Faculty of Computing, Harbin Institute of Technology, Harbin, Heilongjiang 150001, China; Zhengzhou Research Institute, Harbin Institute of Technology, Zhengzhou, Henan 450000, China

## Abstract

**Summary:**

Nanopore sequencing technology enables real-time sequencing and is widely used in rapid detection applications. However, in clinical scenarios, existing structural variant (SV) detection tools typically separate sequencing from computation, limiting their timeliness for clinical applications. To address this, we introduce cuteSV-OL, a novel framework designed for real-time SV discovery, which can be embedded within nanopore sequencing instruments to analyze data concurrently with its generation. Additionally, cuteSV-OL features a real-time SV detection rate evaluation module, allowing users to terminate sequencing early when appropriate, thereby reducing time and cost. Experimental results show that on a standard desktop computer, cuteSV-OL can perform real-time analysis during sequencing and complete SV calling within min after sequencing ends, achieving performance comparable to offline methods. This approach has the potential to enhance rapid clinical diagnostics.

**Availability and implementation:**

cuteSV-OL is released under the MIT license and is available at https://github.com/gwmHIT/cuteSV-OL. It can also be installed via Bioconda or accessed through https://doi.org/10.5281/zenodo.17777436.

## 1 Introduction

Nanopore sequencing technology, characterized by long reads and real-time sequencing, has become one of the most widely used technologies in cutting-edge genomics, clinical diagnostics, and related fields (Jain *et al.* 2016). Compared with conventional second-generation sequencing, the long-read advantage of nanopore sequencing has significantly improved the detection of genomic structural variations (Sedlazeck *et al.* 2018). Currently, structural variation (SV) detection algorithms developed for nanopore sequencing include cuteSV (Jiang *et al.* 2020, 2025), Sniffles2 (Smolka *et al.* 2024), SVIM (Heller and Vingron 2019), and others. These methods collect complete sequencing data and perform sequence alignment, signal extraction, and feature clustering to detect structural variations, contributing significantly to understanding the distribution of human genomic SVs and their relationships to health and disease (Ahsan *et al.* 2023).

However, these approaches follow a sequential sequencing-then-computation model—i.e. sequencing is performed first, followed by analysis—making it challenging to meet the rapid turnaround required for clinical diagnostics and other real-time applications. This delay hinders the timely identification of critical variant information. Some attempts have been made to address this issue. For instance, Goenka *et al.* (2022) proposed accelerating sequencing output by stacking sequencing instruments and scaling up computational facilities, as well as reducing data transmission times via cloud storage. Liu *et al.* (2021) proposed skeleton-based analysis toolkit for SV detection, that applied alignment skeletons as input enabling fast read mapping. However, these methods do not fundamentally depart from the separated sequencing-and-computation model and impose high costs, making them impractical for users with limited sequencing and computing resources.

A more efficient solution would be to integrate real-time data analysis with the sequencing process. In this paradigm, computation would accompany sequencing, enabling the analysis of one batch of sequencing data while the next batch is being generated. This overlapping workflow would allow the data analysis process to nearly coincide with data production, significantly accelerating results even on a single sequencing device paired with a standard edge computing system.

To this end, we propose cuteSV-OL, a real-time structural variation detection framework for nanopore sequencing devices. This method performs real-time sequence alignment and variant signal feature extraction on each batch of data generated by the sequencer, while simultaneously clustering accumulated variant features and detecting SVs in real time. Additionally, it incorporates a real-time SV detection rate evaluation module, which compares detected SVs with a user-defined set and provides feedback on whether sequencing can be terminated early, thereby reducing time and cost. Furthermore, cuteSV-OL features fault recovery mechanisms that restore analytical processes in case of unexpected shutdowns, ensuring data integrity. This approach can be seamlessly integrated into mainstream desktop systems alongside nanopore sequencers, offering a high-efficiency solution for time-sensitive clinical diagnostics.

## 2 Methods

**Figure 1.**
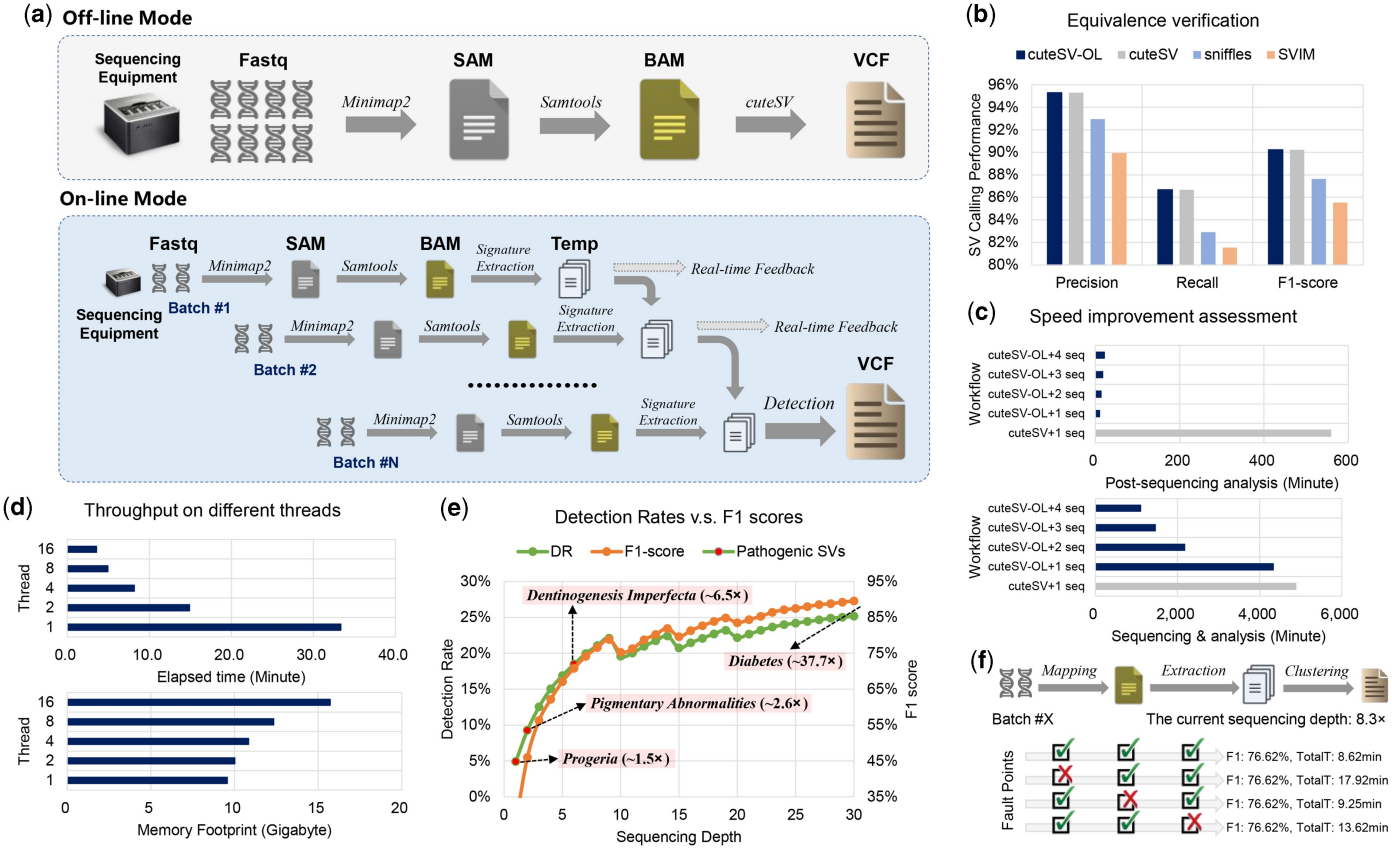
Overview of cuteSV-OL and its benchmarking results. (a) Implementation of cuteSV-OL in On-line Mode and comparison with Off-line Mode. (b) Equivalence verification of detection performance for cuteSV-OL and other tools on a 45× ONT sequencing data for the HG002 human sample. (c) Benchmark results of the analysis time for on-line and off-line modes, under post-sequencing and the whole process respectively. In off-line mode, the post-sequencing analysis time is primarily attributable to the alignment overhead of minimap2. The “cuteSV-OL+x seq” means cuteSV-OL applied x ONT P48 sequencing chips that are involved in real-time sequencing. (d) The throughput of elapsed time and memory footprint of cuteSV-OL under different threads on a sequencing batch. (e) The comparison of the detection rates (main *y*-axis) used for early sequencing termination and F1 scores (secondary *y*-axis) on the HG002 human sample, under different sequencing coverages. The “detection rate” represents the percentage of this population-based ground truth (SVs with AF > 0.1 from HGSVC) that was successfully identified in the HG002 sample by cuteSV-OL and its periodic declines are attributable to adjustments of the support read setting according to sequencing depth. Red dots denote the detection depths of four selected known pathogenic SVs. (f) Performance comparison of SV calling of cuteSV-OL after undergoing different fault point treatments, where a fault point refers to a failure event that causes the pipeline to halt. TotalT indicates the total execution time required to produce the correct callsets for the current batch.

cuteSV-OL is a redeveloped version of the widely used offline SV detector cuteSV. A typical cuteSV-OL processing workflow consists of four key steps (Fig. 1a):

- *File monitoring and task queue management:* cuteSV-OL uses a file monitoring watchdog to continuously monitor incoming FASTQ files. Once a file stabilizes, it is automatically added to the task queue.
- *Preliminary SV signature extraction:* cuteSV-OL processes each FASTQ file in the task queue sequentially. It utilizes minimap2 (Li 2018) for sequence alignment, samtools (Li *et al.* 2009) for BAM file processing, and the cuteSV signal extraction module to obtain preliminary SV signatures. Simultaneously, pandepth (Yu *et al.* 2024) is used to measure the sequencing depth of each FASTQ file.
- *SV clustering and genotyping:* Once all current FASTQ files have been converted into SV signatures, cuteSV-OL applies the cuteSV clustering module to cluster and genotype these signatures. This process considers the current sequencing depth to generate a temporary VCF file containing the SV detection results.
- *Real-time feedback:* Finally, cuteSV-OL uses a detection rate evaluation module to compare the detected SVs with built-in cohort-level SV callsets (or user-defined SV callsets). It calculates the coverage of high-frequency variants as an evaluation metric, providing real-time feedback on sequencing progress.

For additional details, please refer to the Supplementary Methods, available as supplementary data at *Bioinformatics* online.

## 3 Results

To evaluate the real-time SV detection performance of cuteSV-OL, we simulated an actual nanopore sequencing run using 45× HG002 sequencing data from the Oxford Nanopore Technologies platform (ONT). To accurately mimic the batch-wise production of sequencing data, we set the total sequencing time to 72 h, which is the typical runtime for an ONT PromethION platform. Next, we divided the original 45× FASTQ file into multiple non-overlapping files, each containing an equal number of reads (*N* = 60 000). These FASTQ files were then sequentially fed into cuteSV-OL at fixed time intervals (13.5 min), calculated as the total sequencing duration divided by the number of files.

### 3.1 Assessment of the SV detection performance of cuteSV-OL

Ensuring equivalent detection performance is a prerequisite for real-time SV detection. To verify that the SV detection performance of cuteSV-OL matches that of traditional offline method (i.e. cuteSV), we compared the HG002 VCF files generated by each approach against the ground truth set from GIAB (Zook *et al.* 2020). And to obtain a more comprehensive evaluation, we also included two widely used SV detection methods, Sniffles2 and SVIM, in the comparison. The results, presented in Fig. 1b and Table 1, available as supplementary data at *Bioinformatics* online, show that cuteSV-OL achieved identical precision, recall and F1-score compared to cuteSV while also attaining higher accuracy and sensitivity relative to the other two methods. These findings confirm that our proposed real-time cuteSV-OL method maintains detection performance without any loss and further demonstrate that it remains one of the state-of-the-art algorithms for SV detection.

### 3.2 Speed improvement assessment

To evaluate the speed improvement of cuteSV-OL compared to traditional offline methods (only cuteSV retained for the following assessments), we measured both the total sequencing and analysis time (termed total-time) and the post-sequencing analysis time (termed analysis-only-time) required to process 45× HG002 sequencing data under three scenarios: offline mode, single-sequencing chips online mode, and multi-sequencing chips online mode. As shown in Fig. 1c and Table 2, available as supplementary data at *Bioinformatics* online, the single-sequencing chips online mode reduced total-time and analysis-only-time by >11% and 98%, respectively, compared to the offline mode. Given that sequencing accounts for the majority of time consumption (approximately 90%), the total-time for cuteSV-OL further decreased exponentially as the number of sequencing chips increased. Thus, cuteSV-OL significantly accelerates SV detection by drastically reducing post-sequencing analysis time while maintaining detection accuracy. Moreover, its remarkable scalability across multiple sequencing chips highlights its potential for achieving clinical-grade real-time SV detection.

### 3.3 Throughput of cuteSV-OL across different thread configurations

While the online mode of cuteSV-OL offers substantial advantages, an optimal strategy for computational resource allocation to effectively manage multiple sequencing chips remains uncertain. To address this, we systematically analyzed the relationship between processing time per batch and batch interval across different thread configurations (Fig. 1d and Table 3, available as supplementary data at *Bioinformatics* online). Our results show that under the current sequencing settings (batch interval = 13.5 min), cuteSV-OL requires only four threads to process each batch before the arrival of the next one, ensuring a throughput greater than the sequencing output rate. As the number of threads increases, the processing time per batch further decreases—at 16 threads, it accounts for only 27% (i.e. 3.57 min) of the batch interval. This leaves substantial computational headroom to accommodate sequencing data from additional instruments. Notably, increasing the thread count does not significantly impact memory usage, demonstrating that cuteSV-OL can efficiently run on standard desktop computers while managing real-time sequencing data from multiple sequencing chips.

### 3.4 cuteSV-OL adapts to variable sequencing rates

In real ONT sequencing workflows, the rate of read generation is inherently variable. To evaluate the ability of cuteSV-OL to handle reads produced at fluctuating rates, we used the ONT sequencing simulator Icarust (https://github.com/LooseLab/Icarust), which generates raw read signals at variable rates, followed by real-time basecalling conducted with Dorado (https://github.com/nanoporetech/dorado) and structural variant detection conducted with cuteSV-OL. We recorded the cumulative number of reads generated and processed over time (Fig. 1, available as supplementary data at *Bioinformatics* online). The results demonstrated that the processing speed of cuteSV-OL closely matched the rate of read generation, indicating that cuteSV-OL is capable of operating effectively under the fluctuating sequencing rates, thereby maintaining stability during extended sequencing runs.

### 3.5 cuteSV-OL enables early sequencing termination

Real-time nanopore sequencing enables on-demand data production, allowing sequencing depth to be tailored to specific user requirements. To leverage this advantage, we developed a real-time feedback module in cuteSV-OL, which continuously evaluates SV detection performance and provides guidance on when sequencing can be stopped. Specifically, we use the detection rate as the evaluation metric for SV detection performance, which refers to the coverage of the user-defined ground truth set by the call set generated by cuteSV-OL. To validate this feature, we used human common SVs from the HGSVC dataset (Ebert *et al.* 2021) (minor allele frequency > 0.1) as the ground truth and calculated the detection rate for HG002 at various sequencing depths. Notably, we also included corresponding F1 scores at each sequencing depth for auxiliary reference, which were calculated using the ground truth set of HG002 itself provided by GIAB. The results indicate that the detection rates increase rapidly at lower sequencing depths but gradually plateau as depth increases (Fig. 1e and Table 4, available as supplementary data at *Bioinformatics* online). Moreover, we introduced four typically known clinically pathogenic SVs and recorded the sequencing depths while detecting them, to further reveal the down-sampling capability of cuteSV-OL for function-associated SVs. In Fig. 1e, most of the pathogenic SVs (3/4) could be detected at relatively early sequencing depths (Table 5, available as supplementary data at *Bioinformatics* online). These suggest that achieving satisfactory SV detection does not require excessively high sequencing depths. By dynamically monitoring detection performance, the real-time feedback module in cuteSV-OL enables early sequencing termination once predefined detection thresholds are met. This capability is particularly advantageous for cost-sensitive applications, such as disease screening or clinical diagnostics, where reducing sequencing time translates into lower costs without compromising accuracy.

### 3.6 cuteSV-OL enables recovery from fault states

The SV calling process involved in this study requires substantial computational resources and time, making unexpected interruptions costly. Restarting the analysis due to failures can be impractical, particularly in real-time sequencing environments. To mitigate this risk, we developed a fault recovery module for cuteSV-OL, ensuring seamless restoration from a fault state without requiring a full restart. To assess its effectiveness, we simulated multiple unexpected downtime scenarios (Fig. 1f and Table 6, available as supplementary data at *Bioinformatics* online) and compared the detection accuracy and computational overhead between the recovery workflows (where cuteSV-OL resumes operation after an interruption) and an uninterrupted workflow (where no failure occurs). The results demonstrate that cuteSV-OL’s fault recovery module successfully restores system functionality without any loss of detection accuracy. Additionally, the recovery time is negligible relative to restart (no >18 min), ensuring minimal disruption to ongoing SV detection. This feature makes cuteSV-OL highly robust for long-duration sequencing runs, reducing the risk of data loss and improving overall operational efficiency in real-time SV detection.

## 4 Discussion

In this work, we propose cuteSV-OL, an SV detection framework that processes sequencing data in batches using a pipelined approach while cumulatively analyzing the results. By leveraging batch processing, cuteSV-OL integrates sequencing, alignment, and signal extraction into a streamlined pipeline. This overlapping of computational steps significantly accelerates SV detection, resulting in a multiplicative improvement in processing speed without compromising detection accuracy. Additionally, cuteSV-OL is designed to operate on resource-limited platforms, such as edge devices and laptops, making it highly accessible. Its real-time analysis capability allows for earlier SV detection and enables users to terminate sequencing when appropriate, effectively reducing sequencing costs. Furthermore, cuteSV-OL can operate efficiently under fluctuating sequencing rates and incorporate fault recovery mechanisms, thereby ensuring adaptability and stability during extended sequencing runs. Despite its advantages, cuteSV-OL has certain limitations. It is highly coupled with cuteSV, preventing it from incorporating features from other SV callers. However, since cuteSV is one of the most advanced SV callers worldwide, this coupling still provides highly desirable SV detection performance, and the online detection concept introduced in this work can be easily integrated into other SV detection tools. In addition, the reference metric “detection rate” proposed in this study to evaluate real-time detection performance is inherently influenced by the quality and representativeness of the reference population. For example, individuals from different populations exhibited substantial variation in detection rate (Table 7, available as supplementary data at *Bioinformatics* online), which could pose challenges for practical application. However, as more comprehensively resolved and diverse population reference datasets become available in the future, this metric is expected to provide a more accurate and reliable assessment of real-time SV detection effectiveness. We believe that this real-time SV detection approach will play a crucial role in future structural variation studies and clinical applications.

## Supplementary Material

btaf668_Supplementary_Data

## Data Availability

cuteSV-OL was implemented in Python and can be easily installed via Bioconda. Its source code is available at https://github.com/gwmHIT/cuteSV-OL under the MIT open-source license. The specific cuteSV-OL release used in this study has been deposited on Zenodo (DOI: https://doi.org/10.5281/zenodo.17777436) (Guo 2025). All data used for benchmarking in this manuscript are provided in Table 8, available as supplementary data at *Bioinformatics* online.
