# Supplementary material for: cuteSV-OL: a real-time structural variation detection framework for nanopore sequencing devices
Source: Bioinformatics. 2025 Dec 17;42(1):btaf668. doi: 10.1093/bioinformatics/btaf668 (PMC12777969; doi:10.1093/bioinformatics/btaf668)
Supplement: btaf668_Supplementary_Data [file btaf668_supplementary_data.zip › cuteSV-OL Supplementary Notes.docx]

**Supplementary Materials**

cuteSV-OL: a real-time structural variation detection framework

**for nanopore sequencing devices**

Weimin Guo^1, +^, Yadong Liu^1, 2, +^, Yadong Wang^1, 2, *^ and Tao Jiang^1, 2, *^

^1^Center for Bioinformatics, Faculty of Computing, Harbin Institute of Technology, Harbin, Heilongjiang 150001, China

^2^Zhengzhou Research Institute, Harbin Institute of Technology, Zhengzhou, Henan, 450000, China

*To whom correspondence should be addressed.

^+^These authors should be regarded as Joint First Authors.

Contents

[Supplementary Figure 1. Evaluating cuteSV-OL performance under Icarust simulations. 3](#_Toc210510451)

[Supplementary Figure 2. Modify the cuteSV workflow to adapt to the online mode. 4](#_Toc210510452)

[Supplementary Table 1. The benchmark results on cuteSV-OL and other state-of-the-art SV callers using a 45× ONT sequencing data. 5](#_Toc210510453)

[Supplementary Table 2. The benchmark results of the speed improvements on a 45× HG002 sample. 6](#_Toc210510454)

[Supplementary Table 3. Throughput and memory of cuteSV-OL across different thread configuration. 7](#_Toc210510455)

[Supplementary Table 4. The relation between Sequencing depth and detection performance. 8](#_Toc210510456)

[Supplementary Table 5. The benchmark of pathogenic structural variants detection. 9](#_Toc210510457)

[Supplementary Table 6. The benchmark of cuteSV-OL fault recovery performance. 10](#_Toc210510458)

[Supplementary Table 7. Benchmark of common SV detection rates in other samples. 11](#_Toc210510459)

[Supplementary Table 8. Data availability. 12](#_Toc210510460)

[Supplementary Table 9. The benchmark of cuteSV-OL codes optimizations using Cython. 13](#_Toc210510461)

[Supplementary Notes 14](#_Toc210510462)

[1. Supplementary Methods 14](#_Toc210510463)

[1.1 The concrete realization of cuteSV-OL framework 14](#_Toc210510464)

[1.2 Split and improve cuteSV to fit batch detection methods 14](#_Toc210510465)

[1.3 cuteSV-OL can reduce sequencing depth 15](#_Toc210510466)

[2. Implementation of Benchmarking 15](#_Toc210510467)

[2.1 Simulation of real sequencing process 15](#_Toc210510468)

[2.2 Evaluation of cuteSV-OL callsets 16](#_Toc210510469)

[2.3 The benchmark on speed of cuteSV-OL 16](#_Toc210510470)

[2.4 The benchmark of real-time feedback module and fault recovery module 17](#_Toc210510471)

[2.5 The benchmark of pathogenic structural variants detection 17](#_Toc210510472)

[2.6 The benchmark of cuteSV-OL codes optimizations using Cython. 17](#_Toc210510473)

[2.7 Evaluating cuteSV-OL performance under Icarust simulations 18](#_Toc210510474)

[2.8 The command lines for benchmarking 18](#_Toc210510475)

[References 20](#_Toc210510476)


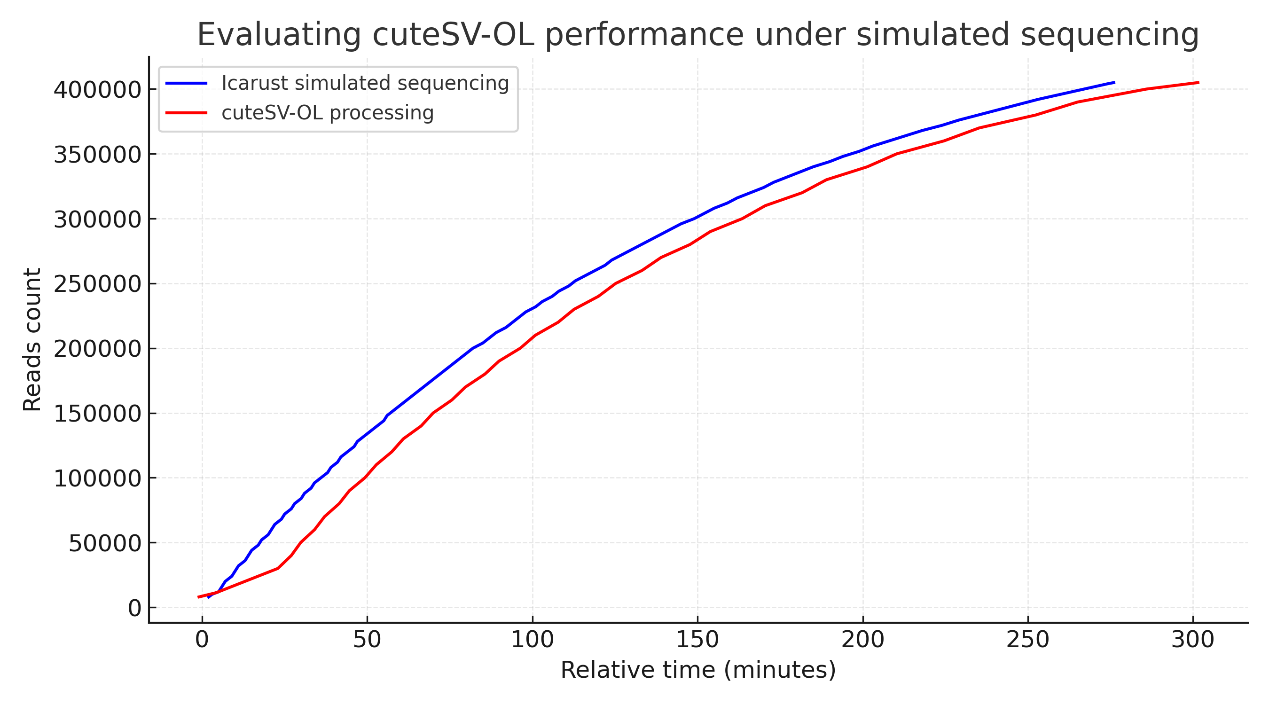


**Supplementary Figure 1. Evaluating cuteSV-OL performance under Icarust simulations.**

We used Icarust, the ONT sequencing simulator you recommended, to generate reads, with the parameter set to output one POD5 file per 4,000 reads (the recommended setting in Icarust). We continuously monitored the output directory, and as soon as a POD5 file was generated, it was passed to Dorado for basecalling. Using pipelines combined with seqkit, the Dorado output was split into FASTQ chunks, each containing 10,000 reads (To balance the overhead of initiating Dorado). cuteSV-OL monitored the FASTQ directory and processed each chunk in real time. The total number of simulated reads was 400,000. We further recorded the cumulative number of reads generated by Icarust and the cumulative number processed by cuteSV-OL over time. As shown in the figure below, cuteSV-OL was able to keep up with the rate of read generation after a certain delay. Detailed experimental procedures can be found in Supplementary Notes 2.7.


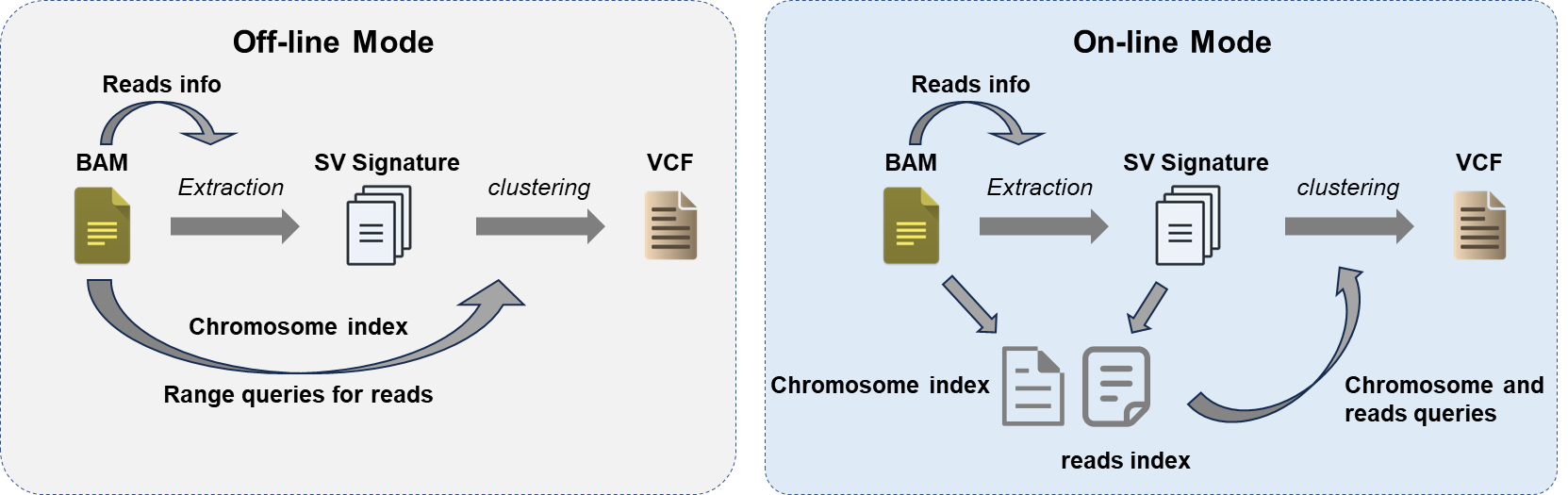


**Supplementary Figure 2. Modify the cuteSV workflow to adapt to the online mode.**

In the original cuteSV pipeline, both the signature extraction and SV clustering modules depend on accessing the BAM file to retrieve information, such as the chromosome index, and to perform range queries for read counts. In real-time SV detection, the complete BAM file is not available, so the necessary information must be extracted from fragmented BAM files. To optimize for a real-time environment, cuteSV-OL decouples the SV clustering step from the BAM file. The BAM file is now used exclusively during the initial signature extraction stage. We pre-store the chromosome index and reads index information in a separate, lightweight file. The SV clustering module uses this file, along with the positional data already contained within the SV Signatures themselves, to perform range queries for chromosome and reads, eliminating the need to query a large BAM file.

1. The benchmark results on cuteSV-OL and other state-of-the-art SV callers using a 45× ONT sequencing data.

| **Tools** | **Precision** | **Recall** | **F1 score** |
| --- | --- | --- | --- |
| CuteSV | 95.29% | 86.67% | 90.23% |
| CuteSV-OL |  |  |  |
| Sniffles2 | 92.95% | 82.91% | 87.64% |
| SVIM | 89.93% | 81.55% | 85.54% |

The evaluation results were computed by Truvari.

1. The benchmark results of the speed improvements on a 45× HG002 sample.

| **Mode** | **Post-sequencing analysis time (min)** | **Sequencing & analysis time (min)** | **F1 score** |
| --- | --- | --- | --- |
| **CPU threads = 16** | | | |
| cuteSV with 1 seq-chip | 558 | 4,878 | 90.23% |
| cuteSV-OL with 1 seq-chip | 9.85 | 4,330 | 90.23% |
| cuteSV-OL with 2 seq-chip | 13.53 | 2,174 | 90.23% |
| cuteSV-OL with 3 seq-chip | 17.18 | 1,457 | 90.23% |
| cuteSV-OL with 4 seq-chip | 21.38 | 1,101 | 90.23% |

The seq-chip represent the sequencing chip for ONT PromethION platforms. The evaluation results were computed by Truvari.

1. Throughput and memory of cuteSV-OL across different thread configuration.

| **Thread number** | **Elapse time (min)** | **Memory (GB)** |
| --- | --- | --- |
| 1 | 33.35 | 9.57 |
| 2 | 14.92 | 10.02 |
| 4 | 8.18 | 10.85 |
| 8 | 4.95 | 12.35 |
| 16 | 3.57 | 15.73 |

1. The relation between Sequencing depth and detection performance.

| **Depth** | **Supporting reads** | **Detection rate** | **F1 score** |
| --- | --- | --- | --- |
| 1 | 2 | 4.91% | 27.65% |
| 2 |  | 9.26% | 46.01% |
| 3 |  | 12.54% | 56.28% |
| 4 |  | 15.04% | 62.20% |
| 5 |  | 16.89% | 67.10% |
| 6 |  | 18.49% | 70.85% |
| 7 |  | 19.96% | 74.13% |
| 8 |  | 21.08% | 76.62% |
| 9 |  | 22.11% | 78.84% |
| 10 | 3 | 19.59% | 75.28% |
| 11 |  | 20.04% | 76.27% |
| 12 |  | 20.98% | 78.74% |
| 13 |  | 21.74% | 80.24% |
| 14 |  | 22.42% | 81.94% |
| 15 | 4 | 20.76% | 79.64% |
| 16 |  | 21.46% | 81.32% |
| 17 |  | 22.09% | 82.67% |
| 18 |  | 22.70% | 83.85% |
| 19 |  | 23.21% | 84.84% |
| 20 | 5 | 22.19% | 83.50% |
| 21 |  | 22.67% | 84.39% |
| 22 |  | 23.21% | 85.28% |
| 23 |  | 23.65% | 86.43% |
| 24 |  | 23.99% | 87.11% |
| 25 |  | 24.20% | 87.49% |
| 26 |  | 24.43% | 87.99% |
| 27 |  | 24.65% | 88.54% |
| 28 |  | 24.88% | 88.81% |
| 29 |  | 25.02% | 89.24% |
| 30 |  | 25.16% | 89.60% |

The F1 results were computed by Truvari. The detection rates were calculated by in-house scripts, which is available at <https://github.com/gwmHIT/cuteSV-OL/blob/master/src/online/compare_model.py>. Detailed experimental procedures can be found in Supplementary Notes 2.4.

1. The benchmark of pathogenic structural variants detection.

| **Chr** | **Descriptions** | **SV type** | **detection depth** | **Total depth of the sample** |
| --- | --- | --- | --- | --- |
| 1: 108190698 | *Progeria* | DEL | 1.5 | 45 |
| 2: 237542608 | *Pigmentary abnormalities of the skin and hair* | DEL | 2.6 |  |
| 4: 87615855 | *Dentinogenesis imperfecta* | DEL | 6.5 |  |
| 9: 133071372 | *Diabetes* | DEL | 37.7 |  |

Detailed experimental procedures can be found in Supplementary Notes 2.5.

1. The benchmark of cuteSV-OL fault recovery performance.

| **Fault point** | **F1 (depth = 8.3)** | **Single batch time (min)** |
| --- | --- | --- |
| No fault | 76.62% | 8.62 |
| Fault point I | 76.62% | 17.92 |
| Fault point II | 76.62% | 9.25 |
| Fault point III | 76.62% | 13.62 |

The selected batched includes all three stages: alignment, signature extraction, and feature clustering. The F1 results were computed by Truvari. Detailed experimental procedures can be found in Supplementary Notes 2.4.

1. Benchmark of common SV detection rates in other samples.

| **Sample** | **Population** | **counts in cuteSV-OL** | **Counts in ground truth** | **Detection rate** |
| --- | --- | --- | --- | --- |
| HG002 | EUR | 12,234 | 47,932 | 25.52% |
| HG003 | EUR | 12,101 |  | 25.25% |
| NA19238 | AFR | 16,556 |  | 34.54% |
| HG02666 | AFR | 16,530 |  | 34.49% |
| HG00512 | EAS | 16,215 |  | 33.83% |
| HG00514 | EAS | 15,459 |  | 32.25% |
| HG00731 | AMR | 16,193 |  | 33.78% |
| HG00732 | AMR | 16,472 |  | 34.37% |

The ground truth is the high-frequency variants (allele frequency > 0.1) from the HGSVC SV atlas. Detailed experimental procedures can be found in Supplementary Notes 2.4.

1. Data availability.

| **Dataset** | **Link** |
| --- | --- |
| HG002 ONT fastq.gz | <https://ftp-trace.ncbi.nlm.nih.gov/giab/ftp/data/AshkenazimTrio/HG002_NA24385_son/Ultralong_OxfordNanopore/guppy-V3.4.5/HG002_ONT-UL_GIAB_20200204.fastq.gz> |
| GRCH38 HG002 T2T data | <https://ftp-trace.ncbi.nlm.nih.gov/ReferenceSamples/giab/data/AshkenazimTrio/analysis/NIST_HG002_DraftBenchmark_defrabbV0.019-20241113/GRCh38_HG2-T2TQ100-V1.1_stvar.benchmark.bed>  <https://ftp-trace.ncbi.nlm.nih.gov/ReferenceSamples/giab/data/AshkenazimTrio/analysis/NIST_HG002_DraftBenchmark_defrabbV0.019-20241113/GRCh38_HG2-T2TQ100-V1.1_stvar.vcf.gz> |
| GRCH38 HGSVC population SV INS&DEL | <https://ftp.1000genomes.ebi.ac.uk/vol1/ftp/data_collections/HGSVC3/release/Variant_Calls/1.0/GRCh38/variants_GRCh38_sv_insdel_sym_HGSVC2024v1.0.vcf.gz> |
| GRCH38 reference | <https://www.ncbi.nlm.nih.gov/datasets/genome/GCF_000001405.26> |

1. The benchmark of cuteSV-OL codes optimizations using Cython.

| **Module Name** | **The proportion** | **Improvement proportion** |
| --- | --- | --- |
| Signature extraction module | 42% | 48% |
| SV clustering module | 22% | 25% |
| SV calling process | 100% | 25.6% |

The test results were obtained using LineProfiler.

Supplementary Notes

- 1. Supplementary Methods

1.1 The concrete realization of cuteSV-OL framework

To ensure cross-platform compatibility, cuteSV-OL adopts the Python-based file monitoring library watchdog to detect newly generated sequencing files from nanopore sequencers, instead of using the lower-level and faster Linux inotify API. The performance difference between the two is negligible when monitoring a small number of files. By default, watchdog monitors the creation of fastq files (including their compressed formats) in user-specified directories. Once a new fastq file is detected, its path is recorded into a task queue.

Instead of feeding new files directly into the downstream workflow, cuteSV-OL uses a task queue to buffer pending files. This design choice allows the system to accommodate potential mismatches between sequencing speed and data processing speed. In the downstream workflow, a producer-consumer model is employed to process tasks. Each fastq file is aligned using minimap2 (Li et al., 2009), followed by sorting and conversion with samtools (Li et al., 2009) to generate bam files. The tool pandepth (Yu et al., 2024) is also used to calculate the sequencing depth of individual bam files, providing valuable information for more accurate SV clustering.

After processing by cuteSV’s (Jiang et al., 2020) SV signature extraction module, only the compact SV signature data are retained. The original fastq and bam files are no longer required for subsequent analysis and can be deleted in real time to save storage space if needed.

At user-defined batch intervals, cuteSV-OL invokes cuteSV's feature clustering modules to cluster the currently accumulated SV signatures and generate intermediate VCF files containing SV detection results. These VCF files are then evaluated against a user-specified target SV set (by default, a human common SVs from the HGSVC dataset (Ebert et al. 2021) is used, with a customizable minor allele frequency threshold). Based on the detection rate of target SVs in the current VCF, users can assess whether sequencing should be terminated.

In addition, cuteSV-OL is equipped with a fault recovery module to handle system crashes and runtime failures. It achieves fault recovery by persistently storing the runtime state, including the set of arrived batches, the set of processed batches, and the batch number processed before the failure. Since the cuteSV-OL workflow consists of three stages—sequence alignment, signal extraction, and feature clustering—the fault recovery module provides dedicated handling for failures occurring at each stage. If a failure occurs during the sequence alignment stage, the module checks the integrity of the current batch's FASTQ file format, discards incomplete reads, and re-runs the alignment. If the failure happens during the signal extraction stage, the module discards the extracted signals from the current batch and re-extracts them. If the failure occurs during the clustering stage, the module simply re-runs the clustering process. In the event of a system crash, cuteSV-OL resumes from the last unfinished batch based on the persistently stored runtime state, rather than restarting from the beginning.

1.2 Split and improve cuteSV to fit batch detection methods

cuteSV is one of the most advanced tools for detecting individual structural variants (SVs) globally. In our work, we refactored and optimized it for seamless integration into the cuteSV-OL real-time workflow. Specifically, we decoupled the original cuteSV pipeline into two fully independent modules: an SV signature extraction module and an SV feature clustering module.

In the standard cuteSV workflow, SV signature extraction is followed by feature clustering, with the BAM file being used in both steps. However, in the real-time setting of cuteSV-OL, the two modules operate on different scopes of data—the extraction module processes individual batches, while the feature clustering module operates on accumulated data. As a result, the required BAM files differ: the extraction module uses batch-specific BAM files, whereas the clustering module ideally needs a merged BAM file. Repeatedly merging BAM files at every clustering interval would impose significant time and storage overhead.

Upon further investigation, we found that the feature clustering module only requires specific read-level information from the BAM file. Therefore, a more efficient strategy is to pre-extract and persist the necessary read information during the SV signature extraction step, avoiding BAM merging entirely. To this end, we propose a method that stores the relevant read metadata needed for clustering and genotyping during the signature extraction phase.

This approach is designed based on the internal implementation of cuteSV. For instance, when genotyping TRA-type SVs, cuteSV evaluates whether a breakpoint is supported by split reads. Specifically, it checks if reads mapping across the breakpoint are primary alignments; if not (i.e., they are split reads), they are considered supportive of the breakpoint. If the proportion of such non-primary alignments exceeds a predefined threshold, the TRA is deemed reliable. While cuteSV originally uses the pysam library and BAM file to retrieve reads overlapping a specific region, our method directly utilizes a lightweight read file generated during signature extraction and the indexes of chromosome and reads generated by accumulative SV signature. The lightweight read file stores five attributes per read in the format described by Equation (1):

$$\begin{aligned} \left( pos_{start}, pos_{end}, is_{primary}, query_{name}, chr_{name} \right) \#\left( 1 \right) \end{aligned}$$

Using these representation and indexes, we can efficiently filter reads spanning a target region by *pos_start_* and *pos_end_*, and determine whether they are primary alignments via *is_primary_*. A formalized workflow is shown in Supplementary Figure 2.

Our method makes full use of intermediate data already generated during signature extraction, enabling a faster and functionally equivalent alternative to parsing BAM files with pysam. Furthermore, we reimplemented the core routines of both modules in Cython, achieving an approximate 20% speed improvement in the overall cuteSV process. The details are provide in Supplementary Table 9.

1.3 cuteSV-OL can reduce sequencing depth

cuteSV-OL adopts a mini-batch processing strategy for handling fastq files, continuously accumulating SV signatures and enabling real-time generation of VCF files for evaluation. This design allows users to monitor SV detection performance dynamically throughout the sequencing process.

To support this, we provide a user interface that allows users to specify a target SV set that cuteSV-OL aims to recall. The system continuously reports the coverage rate of the target SV set, enabling users to make informed decisions about whether to terminate sequencing. This mechanism effectively helps to reduce sequencing depth while still ensuring the retrieval of desired variants.

- 1. Implementation of Benchmarking

2.1 Simulation of real sequencing process

We simulated a real-world DNA sequencing and SV detection process using the following steps:
First, we selected the HG002 sample sequenced on the ONT platform at 40× depth as the target for SV detection throughout the evaluation. Using the tool seqkit (Shen et al. 2016), we split the original fastq.gz file into 320 sub-files based on the number of reads (N = 60,000 per batch). Next, in a Bash script, we used the cp command to sequentially send these sub-files to the monitoring folder of cuteSV-OL, simulating the arrival of sequencing data at intervals calculated as (total sequencing duration / number of splits). We assumed that a single sequencing run takes 72 hours. Finally, we started cuteSV-OL for testing.

In real ONT sequencing, reads are generated sequentially as electrical signals, which are converted into nucleotide sequences in FASTQ format only after basecalling. To more faithfully simulate this real-time sequencing process, we employed a MinKnow simulator Icarust (https://github.com/LooseLab/Icarust) to generate read-level electrical signals, followed by real-time basecalling with Dorado. Subsequently, we applied cuteSV-OL for real-time structural variation detection and recorded the time-course curves of both the number of reads generated and the number of reads processed by cuteSV-OL, as shown in Supplementary Figure 1. Refer to subsection 2.8 for the command lines used for the Simulation of batch sequencing process and refer to subsection 2.7 for the detailed procedure of Icarust simulation.

2.2 Evaluation of cuteSV-OL callsets

First, we use GRCh38 as the reference genome to perform alignment and variant detection, generating the results in VCF format. In our experiments, we evaluated VCF-formatted call sets under two scenarios: (i) assessing precision, recall, and F1 score using Truvari (English et al., 2022), and (ii) calculating the detection rate with a custom script. Since the available ground truth contains only insertions and deletions, our evaluation was restricted to these two types of structural variants. In above cases, for insertions and deletions in the call set of structural variants (SVs), a prediction is considered a true positive (TP) if and only if it meets the following criteria:

|  | $\left\{\begin{matrix} \max(comp_{s}-1kbp,base_{s})\leq\min(comp_{e}+1kbp,base_{e}) \\ \min(comp_{L},base_{L})/\max(comp_{L},base_{L})\geq0.7 \\ comp_{t}=base_{t} \end{matrix}\right.$ | (2) |
| --- | --- | --- |

where comp_s_, comp_e_, comp_L_, and comp_t_ indicate start coordinate, stop coordinate, size, and SV class of a prediction, and base_s_, base_e_, base_L_, and base_t_ are starting coordinate, end coordinate, size, and SV class of a SV recorded in the ground truth, respectively. In addition, false positive (FP) refers to predictions that cannot meet the above conditions with any item in ground truths. Similarly, a ground truth SV is determined as a false negative (FN) if and only if there is no SV call satisfies the above conditions with it. Then, based on the above concepts and definitions, precision is defined as

|  | $precision=\frac{TPs}{TPs+FPs}$ | (3) |
| --- | --- | --- |

Corresponding, recall is defined as

|  | $recall=\frac{TPs}{TPs+FNs}$ | (4) |
| --- | --- | --- |

The definition of detection rate is the same as that of recall.

and F1-score is defined as

|  | $F1=\frac{2\times precision\times recall}{precision+recall}$ | (5) |
| --- | --- | --- |

2.3 The benchmark on speed of cuteSV-OL

To evaluate the computational performance of cuteSV-OL under different numbers of CPU processes/threads, we used the performance profiling tool time in the Linux system to monitor CPU usage and memory overhead. It is worth noting that time ensures accurate statistics in multi-process/threaded tasks, as it does not overlook the CPU and memory consumption of child processes/threads spawned by the parent process. Additionally, we recorded the runtime of cuteSV-OL for processing a single batch under different process/thread configurations and used the average runtime as the performance metric. The tests were conducted on a platform with an Intel(R) Xeon(R) Gold 6240 CPU @ 2.60GHz, 32GB of DDR4 RAM dual channel at 2933 MHz, and CentOS Linux 7.

2.4 The benchmark of real-time feedback module and fault recovery module

CuteSV-OL compares the current SV call set with a user-defined SV set in real time to assess detection performance. To validate this approach, we use the population-scale SV detection reference set HGSVC as the user-defined SV set and observe how the detection rate of high-frequency variants (minor allele frequency > 0.1) changes with sequencing depth. This allows us to evaluate whether CuteSV-OL’s real-time feedback module provides a downsampling effect. To give a more intuitive view of the detection performance, we map the SV call sets obtained at various sequencing depths to the ground truth set of HG002 itself from GIAB and use the F1 score at each depth as a reference. To further strengthen our validation and provide a broader perspective, we examined additional samples from both the Genome in a Bottle (GIAB) consortium and the 1000 Genomes Project, encompassing individuals of different geographic and ancestral backgrounds. Specifically, the European (EUR) group in our analysis included HG002 and HG003 from the GIAB Ashkenazi Jewish trio (European ancestry), while the African (AFR), East Asian (EAS), and Admixed American (AMR) groups consisted of representative samples from the 1000 Genomes Project. We used the set of human common SVs (allele frequency > 0.1) from the HGSVC cohort, which includes 65 individuals, as the ground truth, and compared the coverage of these common SVs detected by cuteSV-OL across the samples. The results were summarized in Supplementary Table 7.

To test the fault recovery module, we injected faults at three different points within a single batch processing cycle of cuteSV-OL: before SV signature extraction, after SV signature extraction but before SV feature clustering, and after SV feature clustering. For each scenario, we recorded the time overhead and the F1 score of the final generated call set after this batch under both normal (no fault) and faulty conditions. This evaluation was conducted to assess the correctness and efficiency of the fault recovery module in cuteSV-OL.

2.5 The benchmark of pathogenic structural variants detection

To demonstrate that cuteSV-OL can effectively reduce sequencing depth requirements in clinical scenarios involving the detection of highly pathogenic SVs, we annotated the HG002 call set using AnnotSV (Geoffroy et al. 2018). This analysis identified four highly pathogenic SVs associated with progeria, pigmentary abnormalities of the skin and hair, dentinogenesis imperfecta, and diabetes. We then examined the sequencing depths at which these SVs were detected by cuteSV-OL and found that three of them could be identified at a depth as low as 6.5×. This result indicates that certain pathogenic SVs can be reliably detected at relatively low sequencing depths, thereby reducing the overall sequencing cost. The detailed results are provided in Supplementary Table 5.

2.6 The benchmark of cuteSV-OL codes optimizations using Cython.

To enhance performance, our real-time detection tool, cuteSV-OL, features not only a revised operational framework but also key code optimizations. Specifically, we have rewritten the core components of the signal extraction and SV clustering modules using Cython.

We used the Python performance profiling tool LineProfiler to record the proportion of time consumed by the two modules mentioned above, as well as the proportions after optimization and have added the results as Supplementary Table 9. As shown in the table, these optimizations result in a 50% speedup for the signal extraction module, a 6% speedup for the SV clustering module, and an overall workflow acceleration of approximately 25%. These optimizations improved the real-time performance of cuteSV-OL when processing large-scale sequencing data.

2.7 Evaluating cuteSV-OL performance under Icarust simulations

To better approximate the real-time process of ONT sequencing from read generation to SV calling, we have added the following experimental procedure:

First, we used Icarust, the ONT sequencing simulator Icarust (https://github.com/LooseLab/Icarust), to generate reads, with the parameter set to output one POD5 file per 4,000 reads (the recommended setting in Icarust). We continuously monitored the output directory, and as soon as a POD5 file was generated, it was passed to Dorado for basecalling. Using pipelines combined with seqkit, the Dorado output was split into FASTQ chunks, each containing 10,000 reads (To balance the overhead of initiating Dorado). cuteSV-OL monitored the FASTQ directory and processed each chunk in real time. The total number of simulated reads was 400,000.

We further recorded the cumulative number of reads generated by Icarust and the cumulative number processed by cuteSV-OL over time. As shown in the Supplementary figure 1, cuteSV-OL was able to keep up with the rate of read generation after a certain delay.

2.8 The command lines for benchmarking

Contruction of split fastq.gz with seqkit (version 2.9.0)

*seqkit split2 -p 320 -O split_dir HG002_ONT.fastq.gz*

**fastq file sender implemented using bash script**

*#!/bin/bash*

*src_dir=$1*

*tgt_dir=$2*

*send_rate=$3*

*mkdir -p "$tgt_dir"*

*split_files=($src_dir/*.fq.gz)*

*for file in "${split_files[@]}"; do*

*cp "$file" "$tgt_dir"*

*current_time=$(date "+%Y-%m-%d %H:%M:%S")*

*echo "file $file done time：$current_time"*

*sleep $send_rate*

*done*

*echo "ALL DONE"*

Conduct SV detection in Off-line mode with cuteSV(version 2.1.2)

*export conda_env=online*

*source miniconda3/etc/profile.d/conda.sh*

*conda activate $conda_env*

*echo "START TIME: $(date '+%Y-%m-%d %H:%M:%S')"*

*minimap2 -t 16 -ax map-ont HG002_ONT.fq.gz | samtools sort -o HG002.bam*

*samtools index HG002.bam*

*rm -r cuteSV_work_dir*

*mkdir cuteSV_work_dir*

*cuteSV --genotype HG002.bam GRCh38.fa output.vcf cuteSV_work_dir --threads 32 --min_support 5*

*bgzip -c output.vcf > output.vcf.gz*

*tabix output.vcf.gz*

*truvari bench -b GRCh37_HG2-T2TQ100.INDEL.* *vcf.gz -c output.vcf.gz –includebed GRCh38_HG2-T2TQ100-V1.1_stvar.benchmark.bed -p 0 -r 1000 -passonly*

*echo "END TIME: $(date '+%Y-%m-%d %H:%M:%S')"*

Conduct SV detection in On-line mode with cuteSV-OL(version 1.0.0)

*export MONITORED_DIR=~/data/experiment/monitor_dir/*

*export REFPATH=~/data/hg38/hg38.fa*

*export WORK_DIR=~/data/experiment/work_dir/*

*export OUTPUTVCF=~/data/experiment/output_vcf/*

*export CONDAENV=online*

*bash just_sender.sh split_dir monitor_dir 810 &*

*conda activate CONDAENV*

*cuteSV_ONLINE $MONITORED_DIR $REFPATH $WORK_DIR $OUTPUTVCF*

Conduct population high frequency variation detection in On-line mode with cuteSV-OL(version 1.0.0)

*export MONITORED_DIR=~/data/experiment/monitor_dir/*

*export REFPATH=~/data/hg38/hg38.fa*

*export WORK_DIR=~/data/experiment/work_dir/*

*export OUTPUTVCF=~/data/experiment/output_vcf/*

*export CONDAENV=onlineconda activate $conda_env*

*export THREADS=16*

*export MMI-PATH=~/data/hg38/hg38_ref.mmi*

*export MONITOR_FADE=300*

*export POP_FILE=~/data/HGSVC/GRCH38_HGSVC2024v1.0_insdel.vcf*

*export SV_FREQ=0.1*

*export PCTSIZE=0.9*

*export REF_DIST=1000*

*export TARGET_RATE=25*

*export BATCH_INTERVAL=4*

*conda activate CONDAENV*

*bash just_sender.sh split_dir monitor_dir 810 &*

*cuteSV_ONLINE $MONITORED_DIR $REFPATH $WORK_DIR $OUTPUTVCF --mmi_path $MMI-PATH --threads $THREADS --monitor_fade $MONITOR_FADE –target_set $POP_FILE --sv_freq $SV_FREQ --pctsize $PCTSIZE --ref_dist $REF_DIST --target_rate $TARGET_RATE --batch_interval $BATCH_INTERVAL*

References

English, A.C., Menon, V.K., Gibbs, R.A. et al. Truvari: refined structural variant comparison preserves allelic diversity. Genome Biol 23, 271 (2022).

Huiyang Yu, Chunmei Shi, Weiming He, Feng Li, Bo Ouyang, PanDepth, an ultrafast and efficient genomic tool for coverage calculation, Briefings in Bioinformatics, Volume 25, Issue 3, May 2024, bbae197.

Jiang, T.*, et al.* Long-read-based human genomic structural variation detection with cuteSV. *Genome Biol* 2020;21(1).

Li, H. Minimap2: pairwise alignment for nucleotide sequences. *Bioinformatics* 2018;34(18):3094-3100.

Li, H.*, et al.* The Sequence Alignment/Map format and SAMtools. *Bioinformatics* 2009;25(16):2078-2079.

Peter Ebert et al.Haplotype-resolved diverse human genomes and integrated analysis of structural variation.Science372,eabf7117(2021).

The 1000 Genomes Project Consortium. A global reference for human genetic variation. *Nature* 526, 68–74 (2015).

Véronique Geoffroy, Yvan Herenger, Arnaud Kress, Corinne Stoetzel, Amélie Piton, Hélène Dollfus, Jean Muller, AnnotSV: an integrated tool for structural variations annotation, Bioinformatics, Volume 34, Issue 20, October 2018, Pages 3572–3574.

Wei Shen, et al. "SeqKit: a cross-platform and ultrafast toolkit for FASTA/Q file manipulation." PloS one 11.10 (2016): e0163962.

Zook, J.M., Hansen, N.F., Olson, N.D. et al. A robust benchmark for detection of germline large deletions and insertions. Nat Biotechnol 38, 1347–1355 (2020).
